# Supplementary material for: The Architecture of Inactivated SARS-CoV-2 with Postfusion Spikes Revealed by Cryo-EM and Cryo-ET
Source: Structure. 2020 Nov 3;28(11):1218–1224.e4. doi: 10.1016/j.str.2020.10.001 (PMC7557167; doi:10.1016/j.str.2020.10.001)
Supplement: Document S1. Figures S1–S4 and Tables S1–S3 [file mmc1.pdf]

Structure, Volume *28*

Supplemental Information

The Architecture of Inactivated SARS-CoV-2 with Postfusion Spikes Revealed by Cryo-EM and Cryo-ET

Chuang Liu, Luiza Mendonça, Yang Yang, Yuanzhu Gao, Chenguang Shen, Jiwei Liu, Tao Ni, Bin Ju, Congcong Liu, Xian Tang, Jinli Wei, Xiaomin Ma, Yanan Zhu, Weilong Liu, Shuman Xu, Yingxia Liu, Jing Yuan, Jing Wu, Zheng Liu, Zheng Zhang, Lei Liu, Peiyi Wang, and Peijun Zhang

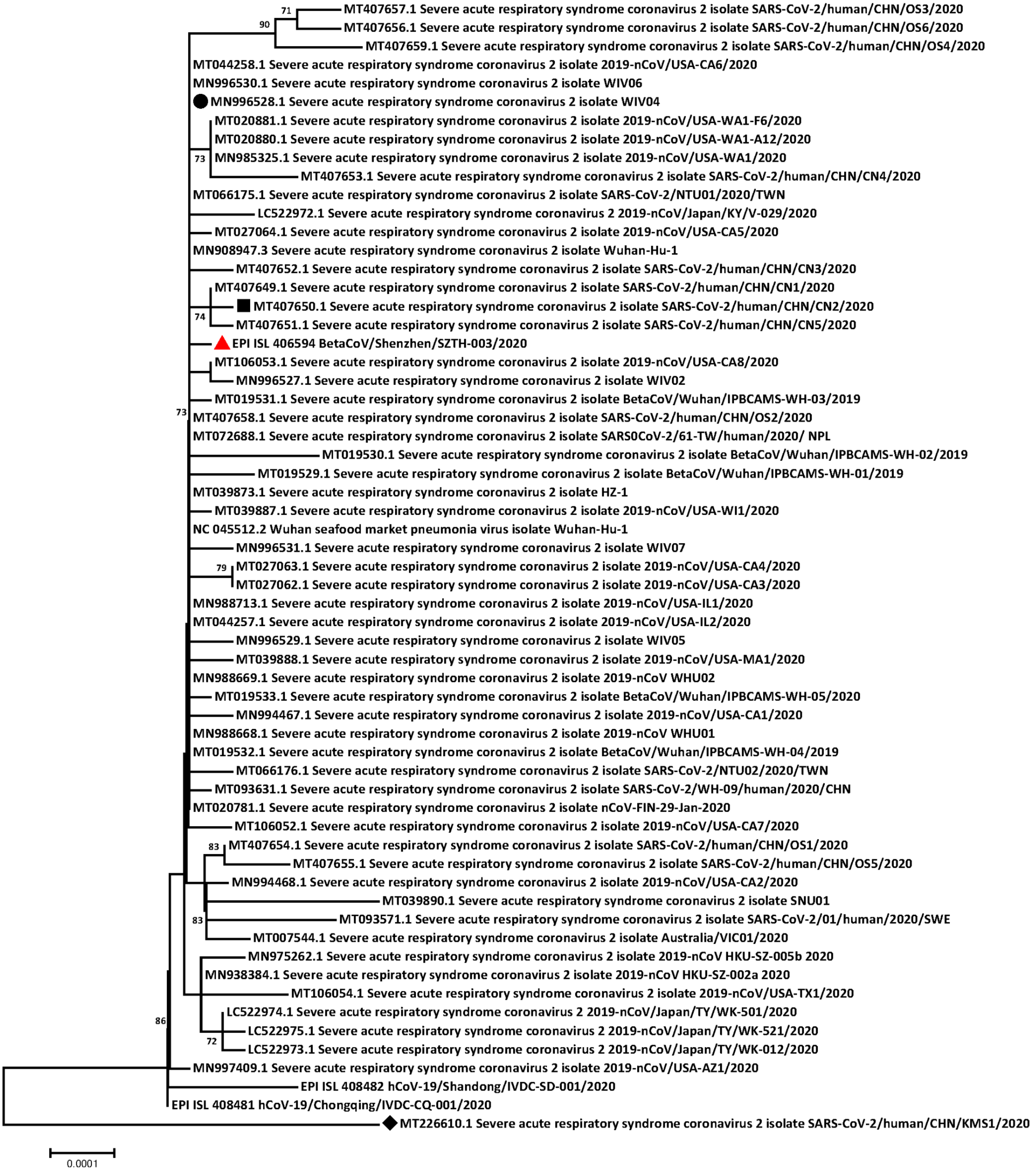

Figure S1 | Phylogenetic characteristics of BetaCoV/Shenzhen/SZTH-003/2020. This isolate was indicated with a triangle. Red triangle, this study; Black dot, WIV04; black squar, CN2; black diamond, UCC91-02. Related to Figure 1.

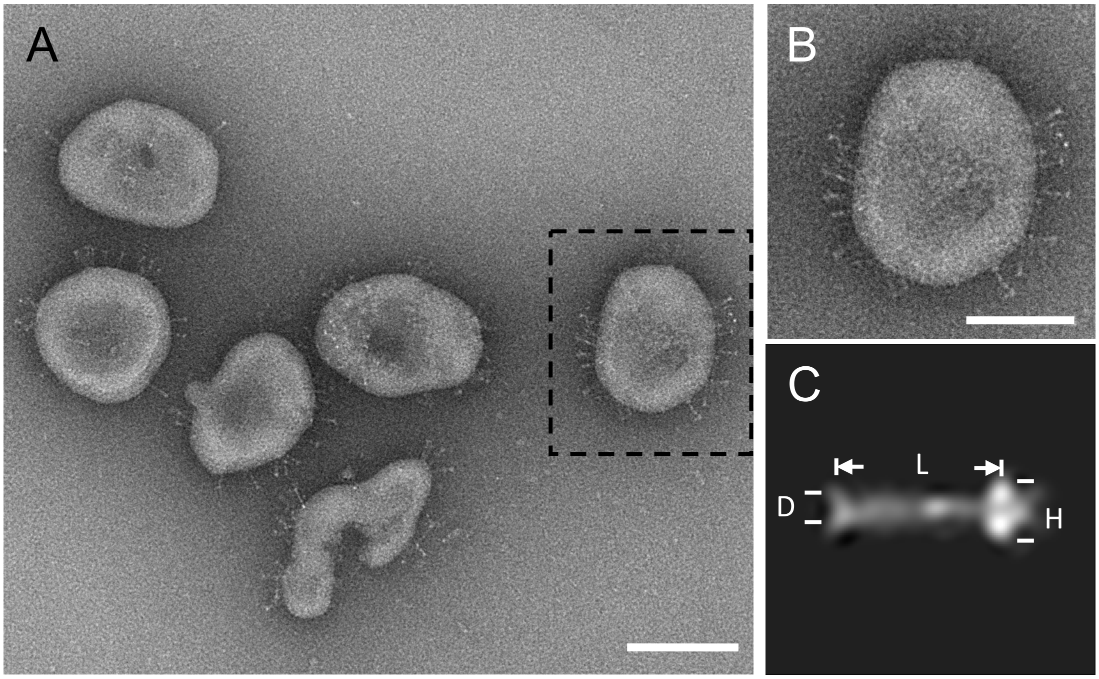

Figure S2 | Characterization of purified SARS-CoV-2 by negative staining EM. (A) Image of negative stained SARS-CoV-2. Nail-like spikes can be clearly seen. (B) Enlarged view of virion boxed in (A). (C) Averaged 2D projection of SARS-CoV-2 nail-shaped spike (Song et al., 2018). The length (L), diameter of stem (D) and spike's head (H) are 23nm, 4nm and 7nm, respectively. Scale bars, 100 nm in A, 50 nm in B. Related to Figure 2.

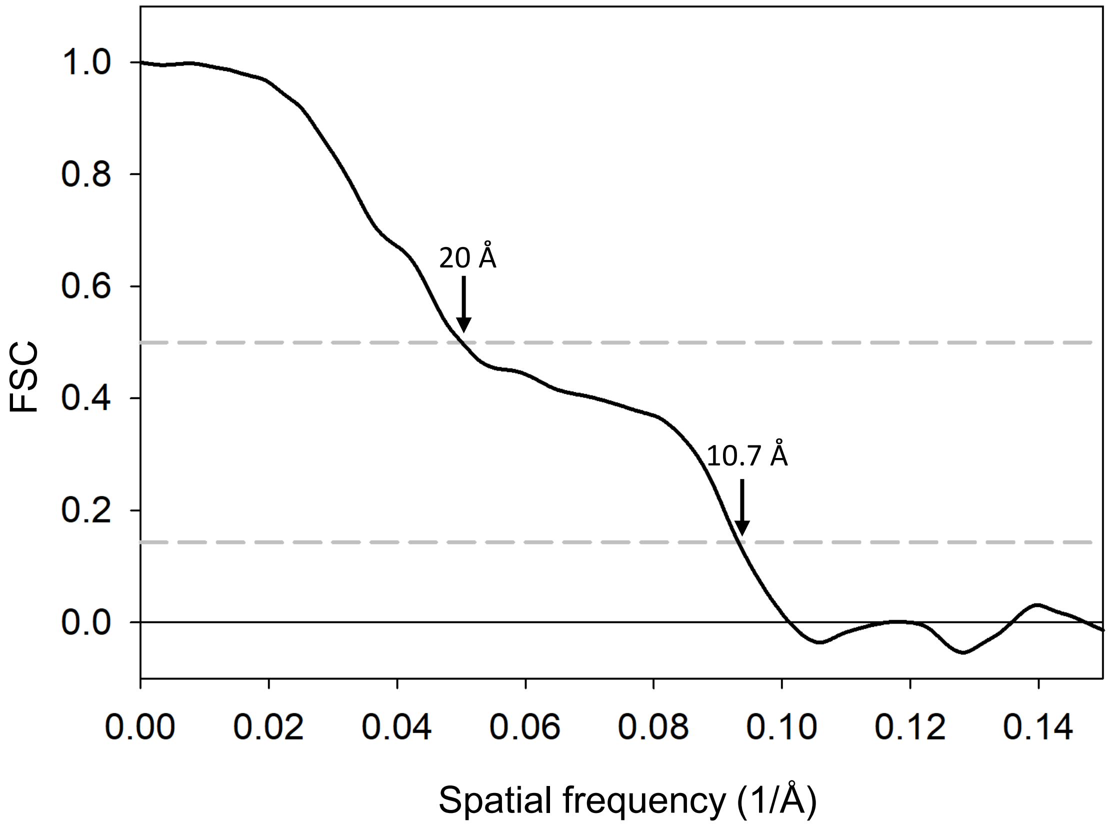

Figure S3 | Fourier-shell-correlation (FSC) plot of postfusion spike subtomogram averaged density maps. The two dashed lines mark the FSC value of 0.5 and 0.143. The resolution is 20 Å at FSC of 0.5 and 10.7 Å at FSC of 0.143. Related to Figure 3.

```
Uniprot|Sequence name

P0DTC2|SPIKE_SARS2      NNSYECDIPIGAGICASYQTQTNSPRRARSVASQSIIAYTMSLGAENSVAYSNNSIAIPT   716
P59594|SPIKE_CVHSA      DTSYECDIPIGAGICASYHTVSL----LRSTSQKSIVAYTMSLGADSSIAYSNNTIAIPT   698
                        :.****************:* :      **.:.:**:********:.*:*****:*****

P0DTC2|SPIKE_SARS2      NFTISVTTEILPVSMTKTSVDCTMYICGDSTECSNLLLQYGSFCTQLNRALTGIAVEQDK   776
P59594|SPIKE_CVHSA      NFSISITTEVMPVSMAKTSVDCNMYICGDSTECANLLLQYGSFCTQLNRALSGIAAEQDR   758
                        **:**:***::****:******.**********:*****************:***.***:

P0DTC2|SPIKE_SARS2      NTQEVFAQVKQIYKTPPIKDFGGFNFSQILPDPSKPSKRSFIEDLLFNKVTLADAGFIKQ   836
P59594|SPIKE_CVHSA      NTREVFAQVKQMYKTPTLKYFGGFNFSQILPDPLKPTKRSFIEDLLFNKVTLADAGFMKQ   818
                        **:********:**** :* ************* **:********************:**

P0DTC2|SPIKE_SARS2      YGDCLGDIAARDLICAQKFNGLTVLPPLLTDEMIAQYTSALLAGTITSGWTFGAGAALQI   896
P59594|SPIKE_CVHSA      YGECLGDINARDLICAQKFNGLTVLPPLLTDDMIAAYTAALVSGTATAGWTFGAGAALQI   878
                        **:***** **********************:*** **:**::** *:************

P0DTC2|SPIKE_SARS2      PFAMQMAYRFNGIGVTQNVLYENQKLIANQFNSAIGKIQDSLSSTASALGKLQDVVNQNA   956
P59594|SPIKE_CVHSA      PFAMQMAYRFNGIGVTQNVLYENQKQIANQFNKAISQIQESLTTTSTALGKLQDVVNQNA   938
                        ************************* ******.**.:**:**::*::*************

P0DTC2|SPIKE_SARS2      QALNTLVKQLSSNFGAISSVLNDILSRLDKVEAEVQIDRLITGRLQSLQTYVTQQLIRAA   1016
P59594|SPIKE_CVHSA      QALNTLVKQLSSNFGAISSVLNDILSRLDKVEAEVQIDRLITGRLQSLQTYVTQQLIRAA   998
                        ************************************************************

P0DTC2|SPIKE_SARS2      EIRASANLAATKMSECVLGQSKRVDFCGKGYHLMSFPQSAPHGVVFLHVTYVPAQEKNFT   1076
P59594|SPIKE_CVHSA      EIRASANLAATKMSECVLGQSKRVDFCGKGYHLMSFPQAAPHGVVFLHVTYVPSQERNFT   1058
                        **************************************:**************:**:***

P0DTC2|SPIKE_SARS2      TAPAICHDGKAHFPREGVFVSNGTHWFVTQRNFYEPQIITTDNTFVSGNCDVVIGIVNNT   1136
P59594|SPIKE_CVHSA      TAPAICHEGKAYFPREGVFVFNGTSWFITQRNFFSPQIITTDNTFVSGNCDVVIGIINNT   1118
                        *******:***:******** *** **:*****:.*********************:***

P0DTC2|SPIKE_SARS2      VYDPLQPELDSFKEELDKYFKNHTSPDVDLGDISGINASVVNIQKEIDRLNEVAKNLNES   1196
P59594|SPIKE_CVHSA      VYDPLQPELDSFKEELDKYFKNHTSPDVDLGDISGINASVVNIQKEIDRLNEVAKNLNES   1178
                        ************************************************************

P0DTC2|SPIKE_SARS2      LIDLQELGKYEQYIKWPWYIWLGFIAGLIAIVMVTIMLCCMTSCCSCLKGCCSCGSCCKF   1256
P59594|SPIKE_CVHSA      LIDLQELGKYEQYIKWPWYVWLGFIAGLIAIVMVTILLCCMTSCCSCLKGACSCGSCCKF   1238
                        *******************:****************:*************.*********

P0DTC2|SPIKE_SARS2      DEDDSEPVLKGVKLHYT        1273
P59594|SPIKE_CVHSA      DEDDSEPVLKGVKLHYT        1255
                        *****************
```

Figure S4 | Sequence alignment between SARS-CoV-2 S protein (Uniprot P0DTC2, Spike_SARS2) and SARS-CoV S protein (Uniprot P59594, Spike_CHHSA), shown in S2 region. Residues in bold indicate S2 residues. Residues in red indicates residues solved in PDB 6M3W. Residues highlighted in green are the glycosylation sites. Alignment done with CLUSTAL Omega (1.2.4). Related to Figure 3.

Table S1. Clinical information of the enrolled patient. Related to Figure 1.

Characteristics	Description
Age	62
Gender	male
Disease severity	severe
Wuhan exposure history	Yes (Jan 1, 2020 – Jan 14, 2020)
Date of symptoms onset	Jan 11, 2020
Initial symptoms	Fever/Cough
Date of hospitalization	Jan 15, 2020
Co-existing chronic disease	none
Influenza A virus	-
Influenza B virus	-
Respiratory syncytial virus	-
Human Bocavirus	-
Adenovirus	-
Human metapneumovirus	-
Rhinovirus	-
HCoV-229E	-
HCoV-OC43	-
HCoV-HKU1	-
HCoV-NL63	-
MERS-CoV	-
SARS-CoV	-
Interferon atomization	Jan 16, 2020
Ribavirin	Jan 15, 2020
Methylprednisolone	no
High-flow oxygen therapy	yes
Mechanical ventilation	No
CT finding	Bilateral pneumonia
BALF sampling date	Jan 21, 2020
Outcome	Discharged

Table S2: Summary of data acquisition and image processing statistics. Related to Figure 2 and 3

<u>Data acquisition</u>	
EM equipment	Titan Krios
Voltage (kV)	300
Detector	Gatan K2
Energy filter	Gatan GIF Quantum, 20 eV slit
Super-resolution mode	Yes
Pixel size (Å)	1.4
Total electron dose (e^-/Å^2)	123
Dose rate (e-/Å^2/s)	2.55
Frame number	10
Acquisition scheme	-60/60°, 3°
Defocus range (µm)	-1.5 to -5.0
Number of tilt-series	383
Software	SerialEM
<u>Data processing</u>	
Number of tomograms	242
Number of subtomograms	956
Symmetry imposed	C3
Final resolution (Å)	20 Å at 0.5FSC, 10.7 Å at 0.143FSC
Software	IMOD/emClarity/Dynamo
EMDB number	EMD-11627

Table S3. Comparison of inactivated viruses from four vaccine candidates with this study. Related to Figure 2 and 3.

Vaccine Name	Developer	Type of Vaccine	Vaccine strain	EM images	reference
PiCoVacc	Sinovac	β-propiolactone	CN2	CryoEM, most postfusion Spikes	Gao et al, Science 2020
N/A	Wuhan Institute of Biological Products/ Sinopharm	β-propiolactone	WIV04	NO EM image	Xia et al. JAMA 2020
BBIBP-CorV	Beijing Institute of Biological Products/ Sinopharm	β-propiolactone	HB02	Negative stain most prefusion Spikes	Wang et al. Cell 2020
N/A	Institute of Medical Biology, Chinese Academy of Medical Sciences	formaldehyde	UCC91-02	Negative stain, both prefusion and post fusion	Chen et al., BioRxiv 2020
N/A	Institute for Hepatology, National Clinical Research Center for Infectious Disease, Shenzhen	β-propiolactone	SZTH-003	Negative stain and cryoEM/ET, both prefusion (26%) and postfusion (74%)	This study
