# Supplementary material for: The Architecture of Inactivated SARS-CoV-2 with Postfusion Spikes Revealed by Cryo-EM and Cryo-ET
Source: Structure. 2020 Nov 3;28(11):1218–1224.e4. doi: 10.1016/j.str.2020.10.001 (PMC7557167; doi:10.1016/j.str.2020.10.001)
Supplement: Document S2. Article plus Supplemental Information [file mmc2.pdf]

Short Article

Structure

The Architecture of Inactivated SARS-CoV-2 with Postfusion Spikes Revealed by Cryo-EM and Cryo-ET

Graphical Abstract

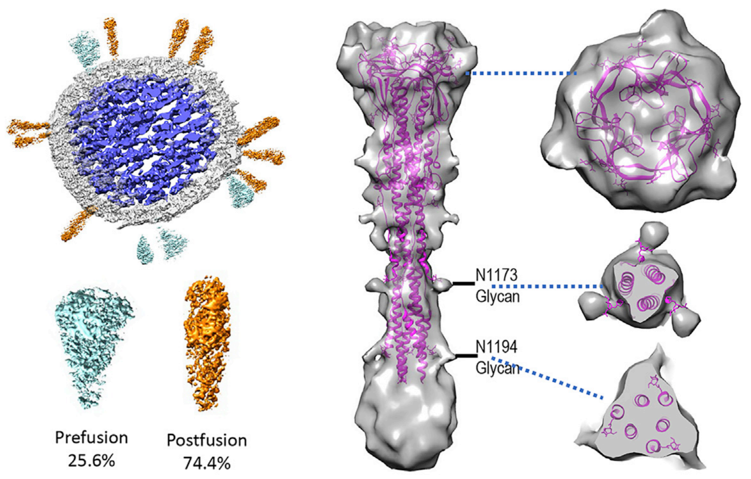

Authors

Chuang Liu, Luiza Mendonça, Yang Yang, ..., Lei Liu, Peiyi Wang, Peijun Zhang

Correspondence

zhangzheng1975@aliyun.com (Z.Z.),
liulei3322@aliyun.com (L.L.),
wangpy@sustech.edu.cn (P.W.),
peijun@strubi.ox.ac.uk (P.Z.)

In Brief

Several vaccine candidates using inactivated SARS-CoV-2 viruses are under development. Liu et al. used state-of-the-art cryoelectron microscopy technologies to characterize the architecture of inactivated SARS-CoV-2 viruses. They found that the viral spikes are mostly in a postfusion state, which is not desirable for vaccine development.

Highlights

- β-propiolactone-inactivated SARS-CoV-2 viruses display postfusion spikes
- Cryo-ET structure of SARS-CoV-2 postfusion spikes was determined at 11 Å resolution
- This study calls for crucial structural characterization of vaccine candidates

Liu et al., 2020, Structure 28, 1218–1224
November 3, 2020 © 2020 The Author(s). Published by Elsevier Ltd.
https://doi.org/10.1016/j.str.2020.10.001

CellPress

Structure

Short Article

The Architecture of Inactivated SARS-CoV-2 with Postfusion Spikes Revealed by Cryo-EM and Cryo-ET

Chuang Liu,[1,4,9] Luiza Mendonça,[2,9] Yang Yang,[3,9] Yuanzhu Gao,[1,4,9] Chenguang Shen,[3,9] Jiwei Liu,[2] Tao Ni,[2] Bin Ju,[3] Congcong Liu,[4,6] Xian Tang,[3] Jinli Wei,[3] Xiaomin Ma,[1,4] Yanan Zhu,[2] Weilong Liu,[3] Shuman Xu,[4] Yingxia Liu,[3,5,6] Jing Yuan,[5] Jing Wu,[4] Zheng Liu,[4] Zheng Zhang,[3,6,*] Lei Liu,[3,6,*] Peiyi Wang,[1,4,*] and Peijun Zhang[2,7,8,*]
[1]Department of Biology, Southern University of Science and Technology, Shenzhen 518055, Guangdong Province, China
[2]Division of Structural Biology, Wellcome Trust Centre for Human Genetics, University of Oxford, Oxford OX3 7BN, UK
[3]Institute for Hepatology, National Clinical Research Center for Infectious Disease, Shenzhen Third People's Hospital, Shenzhen 518112, Guangdong Province, China
[4]Cryo-EM Center, Southern University of Science and Technology, Shenzhen 518055, Guangdong Province, China
[5]Department for Infectious Diseases, Shenzhen Third People's Hospital, Shenzhen, Guangdong Province 518112, China
[6]The Second Affiliated Hospital, School of Medicine, Southern University of Science and Technology, Shenzhen 518055, Guangdong Province, China
[7]Electron Bio-Imaging Centre, Diamond Light Source, Harwell Science and Innovation Campus, Didcot OX11 0DE, UK
[8]Lead Contact
[9]These authors contributed equally
*Correspondence: zhangzheng1975@aliyun.com (Z.Z.), liulei3322@aliyun.com (L.L.), wangpy@sustech.edu.cn (P.W.), peijun@strubi.ox.ac.uk (P.Z.)
https://doi.org/10.1016/j.str.2020.10.001

SUMMARY

The ongoing global pandemic of coronavirus disease 2019 (COVID-19) resulted from the outbreak of SARS-CoV-2 in December 2019. Currently, multiple efforts are being made to rapidly develop vaccines and treatments to fight COVID-19. Current vaccine candidates use inactivated SARS-CoV-2 viruses; therefore, it is important to understand the architecture of inactivated SARS-CoV-2. We have genetically and structurally characterized β-propiolactone-inactivated viruses from a propagated and purified clinical strain of SARS-CoV-2. We observed that the virus particles are roughly spherical or moderately pleiomorphic. Although a small fraction of prefusion spikes are found, most spikes appear nail shaped, thus resembling a postfusion state, where the S1 protein of the spike has disassociated from S2. Cryoelectron tomography and subtomogram averaging of these spikes yielded a density map that closely matches the overall structure of the SARS-CoV postfusion spike and its corresponding glycosylation site. Our findings have major implications for SARS-CoV-2 vaccine design, especially those using inactivated viruses.

INTRODUCTION

Coronaviruses are a large family of zoonotic viruses responsible for multiple large-scale outbreaks in past few decades (Liu et al., 2020; Perlman and Netland, 2009; Yang et al., 2013, 2019). Since early December of 2019, the outbreak of a pneumonia epidemic was determined to be caused by a novel coronavirus (Wang et al., 2020; Zhu et al., 2020), SARS-CoV-2. The resulting disease caused by SARS-CoV-2 was named coronavirus disease 2019 (COVID-19) (Viruses, 2020). As of this writing, more than 11 million confirmed cases of and more than half a million deaths due to COVID-19 have been reported worldwide (WHO, 2020b). Information about the cultivation, purification, and ultrastructure of SARS-CoV-2 in its native state is urgently needed to understand the viral infection process and aid vaccine development and COVID-19 treatment.

Coronaviruses are enveloped single-stranded positive-RNA viruses, roughly 80–120 nm in diameter. The viral RNA is intimately associated with the viral nucleocapsid protein (N), forming large viral ribonucleoprotein complexes. The viral envelope is derived from the host cell and decorated with viral surface proteins: spike (S), membrane (M), and envelope (E).

The viral spike is responsible for cell entry and is the primary target of human antibodies. It is a trimer protein complex composed of S1, responsible for receptor binding, and S2, responsible for viral fusion. S1 and S2 are translated as a single polyprotein that is the target of two proteolytic cleavages. The first is the maturation cleavage in the S1 and S2 boundary by cellular proteases during particle assembly in the endoplasmic reticulum-Golgi transit. The second is the priming (or activation) cleavage, which is performed by the cellular serine protease TMPRSS2 at the S2′ site and is essential for viral entry. It has

 © 2020 The Author(s). Published by Elsevier Ltd.
This is an open access article under the CC BY license (http://creativecommons.org/licenses/by/4.0/).

been shown that both receptor binding and priming cleavages are necessary for cellular entry and viral infectivity (Hoffmann et al., 2020). It is not clear, however, which event triggers the spike conformational change leading to membrane fusion or how receptor binding and the priming cleavage are related.

The prefusion conformations of SARS-CoV-2 spike were determined alone and in complex with the cell surface receptor ACE-2 or a neutralizing antibody (Yan et al., 2020). The structure shows a club-like complex, with S1 occupying most of the exposed surface of the trimer and S2 mostly sequestered. The receptor binding domain (RBD) in S1 is located on the top of the spike, to which ACE-2 binds when it is in the up (or open) conformation (Lan et al., 2020; Yan et al., 2020). There is consensus that the engagement of the S1 RBD with the receptor destabilizes the trimer, triggering the shedding of the S1 units, which allows a remarkable conformational change in the spike from a large club-shaped structure into a thin and long nail-like structure. Nonetheless, *in vitro* trypsin cleavage of recombinant mouse hepatitis virus, SARS-CoV, and Middle East respiratory syndrome coronavirus S proteins also led to the prefusion to postfusion transition, in the absence of receptor binding (Walls et al., 2017). Whereas pre- and postfusion structures of SARS-CoV-2 spike are available from engineered and recombinant proteins, structural studies of the SARS-CoV-2 spike *in situ* in the intact virions are still lacking.

Here we report the successful propagation and purification of SARS-CoV-2 in a BSL-3 laboratory and reveal the whole viral architecture of inactivated SARS-CoV-2 by cryoelectron microscopy and tomography (cryo-EM and cryo-ET). Purified viruses are decorated with viral spikes, most of them adopting a morphology consistent with the postfusion conformation. This opens up the possibility that alternative processes may trigger the S protein conformational change, which has direct relevance to current vaccine development.

RESULTS

Isolation and Identification of the SARS-CoV-2 Virus

A 62-year-old male was admitted to Shenzhen Third People's Hospital on January 15, 2020, with pneumonia and was further diagnosed as COVID-19 positive. An epidemiological investigation confirmed a Wuhan travel history between January 1 and January 14 for this patient, and symptoms began on January 11, 2020, including fever and cough. Testing for common respiratory viruses, including influenza A virus, influenza B virus, adenoviruses, human parainfluenza virus, and other human coronaviruses, was negative. Lymphopenia, elevated C-reactive protein, and elevated interleukin-6 were found upon admission (Table S1). Computed tomography scans showed multiple ground-glass opacities in bilateral lungs at the early stage, and lung consolidation occurred during hospitalization (Figure 1A).

The bronchoalveolar lavage fluid sample was collected and subjected to next-generation sequencing. The genome sequence of this virus was submitted to the Global Initiative on Sharing Avian Flu Data under accession number EPI_ISL_406594 and designated as "BetaCoV/Shenzhen/SZTH-003/2020." Phylogenetic analyses showed that the virus possessed a high homology to two isolates, BetaCoV/Wuhan/IPBCAMS-WH-04/2019 from Wuhan and SARS-CoV-2/NTU01/2020/TWN from Taiwan (Figure S1).

Virus from the patient was propagated and isolated using Vero cells in the BSL-3 (biosafety level 3) laboratory. Typical cytopathic effects were observed 4 days postinoculation in Vero cells, including cell rounding, shrinkage, lysis, and detachment throughout the cell monolayers (Figure 1B). Using the patient's plasma, virus could be detected in cultured cells by immunofluorescence (Figure 1C). Viral RNAs were observed in the cell culture supernatant when probed using a China Food and Drug Administration-approved commercial kit targeting the open reading frame (ORF) 1ab (red in Figure 1D) and N (blue) genes of SARS-CoV-2 with low cycle threshold values (Figure 1D). The purified SARS-CoV-2 particles showed specific reactivity to convalescent plasmas from SARS-CoV-2-infected patients (Figure 1E) and to specific human monoclonal antibodies against the RBD of the S protein using ELISA (Figure 1F). Although both hmAb-1 and hmAb-2 are RBD-specific human monoclonal antibodies, hmAb-1 (named B38) has a relatively higher binding affinity to RBD, with a K_d of 4.48 nM (Wu et al., 2020), compared with hmAb-2 (named P1A-1D1), with a K_d of 260.50 nM (Ju et al., 2020). The ELISA data suggest a certain amount of RBD (or S1 spike) is present in the purified virus particles.

Cryo-EM Analysis of SARS-CoV-2

The isolated and concentrated SARS-CoV-2 virus particles were inactivated with β-propiolactone for structural characterization, initially by negative staining EM followed by cryo-EM and cryo-ET. SARS-CoV-2 displays typical morphology of a coronavirus, where spike proteins decorate the surface of the viral particles (Figure S2A). A close examination of the spikes suggested the presence of two types of spikes; one appears larger, with a club head, resembling the prefusion spike trimer, whereas the other is much thinner and nail shaped, and resembles the postfusion spike containing only the S2 trimer (Song et al., 2018) (Figure S2C). Both types were observed in individual virus particles. Cryo-EM images of purified SARS-CoV-2 virus particles showed that the virus particles are roughly spherical or moderately pleiomorphic, with an average diameter of 108 ± 8 nm, ranging from 84 to 126 nm. The viral spikes and viral membranes are clearly discerned. About 20%–30% of the virions have abundant spikes around the envelope, whereas many other virus particles display few spikes (Figure S2). Consistent with the observation in negative-stain EM, cryo-EM images showed that most spikes are nail shaped, presumably in a postfusion state (Figure 2). Projection images from negative-staining virus particles may not allow for a quantitative measure of the number of spikes. They are, nevertheless, a quick and easy way to assess sample quality. One can already raise a flag if postfusion spikes are observed in negatively stained images, which would prompt a more careful 3D analysis.

Cryo-ET and Subtomogram Averaging of SARS-CoV-2 Postfusion Spike

The 3D nature of cryo-ET allows analysis of features that would normally be obstructed in cryo-EM projection images (Ni et al., 2020; Zhang, 2019). Detailed parameters for tomography data collection and processing are summarized in Table S2. The spikes in the tomograms confirmed the findings from negative

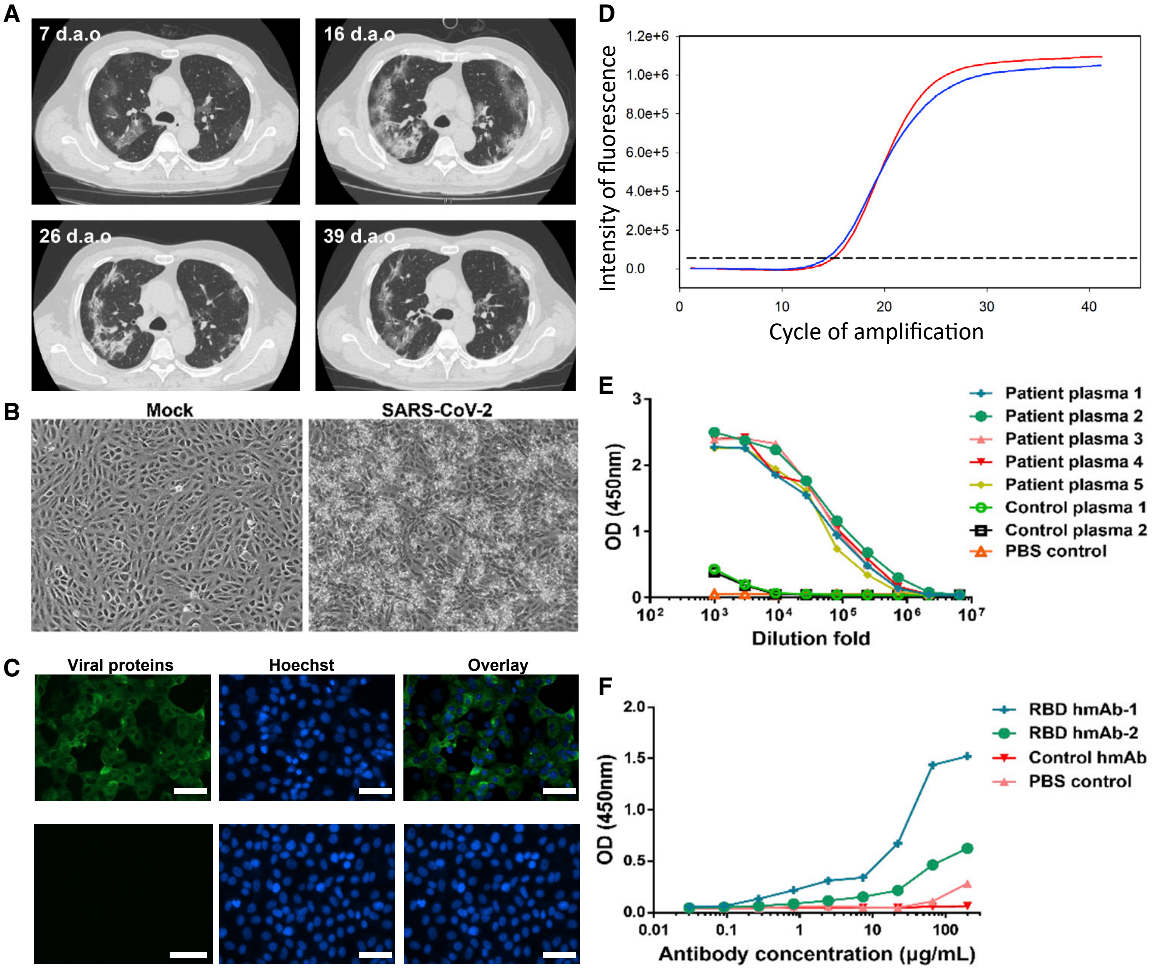

Figure 1. Isolation and Identification of the SARS-CoV-2 Virus

(A) Representative computed tomography scans of the patient at 7, 16, 26, and 39 days after illness onset (d.a.o).

(B) Vero cells were inoculated with a bronchoalveolar lavage fluid sample. The cytopathic effects were observed at 4 days postinfection.

(C) Detection of virus by indirect immunofluorescence assay using the patient's plasma (top) and control plasma from a healthy individual (bottom).

(D) Viral RNAs were extracted from the cell culture supernatant and detected using a commercial kit probing the ORF 1ab (red) and N (blue) genes of SARS-CoV-2.

(E and F) Testing the convalescent plasma IgG antibody (E) and SARS-CoV-2 RBD-specific human monoclonal antibodies (F) using purified SARS-CoV-2 virus particles. The control plasmas 1 and 2 were obtained from a patient recovered from influenza A virus infection and a healthy volunteer, respectively. All data points represent duplicate measurements. The control monoclonal antibody is a human monoclonal antibody specific to influenza A virus generated by the Institute for Hepatology in the Third People's Hospital of Shenzhen. Scale bar, 50 μm.

staining and cryo-EM, showing that the spikes adopt a conformation consistent with the postfusion state of the S2 protein (Figures 3A and 3B). Frequently, the spikes were arranged in clusters (Figures S2B and 2D). Among 125 spikes from 19 virus particles analyzed from high defocused tomograms, we observed 32 prefusion and 93 postfusion spikes from which S1 had dissociated. The postfusion spikes account for 74% of total spikes in the β-propiolactone-treated SARS-CoV-2 particles (Figure 3C). Virus tomograms also showed the ribonucleoprotein complexes organized inside the viruses (Figures 3A–3C).

Despite the determination of many structures of prefusion SARS-CoV-2 spike alone and in complex with its receptor and antibodies, the structure of the postfusion SARS-CoV-2 spike is still lacking. A recent study showed the structure of postfusion SARS-CoV spikes from purified recombinant S proteins using single-particle cryo-EM (PDB: 6M3W) (Fan et al., 2020). To obtain an *in situ* 3D structure of the postfusion SARS-CoV-2 spike on the native virion, we carried out cryo-ET and subtomogram alignment and averaging (Sutton et al., 2020; Zhang, 2019). A set of 956 manually picked nail-shaped spike subvolumes from 242 tomograms were aligned and averaged iteratively with a 3-fold symmetry applied using the programs Dynamo and emClarity (Castaño-Díez et al., 2012; Himes and Zhang, 2018). The resulting map at 11 Å resolution showed the characteristic postfusion conformation of coronavirus spikes (Figures 3D and S3): a 240 Å tall structure, 66 Å at its widest point, where the connector domains and core β sheets are, and 27 Å at its narrowest point, near heptad repeat 1 (HR1). The atomic model of postfusion SARS-CoV

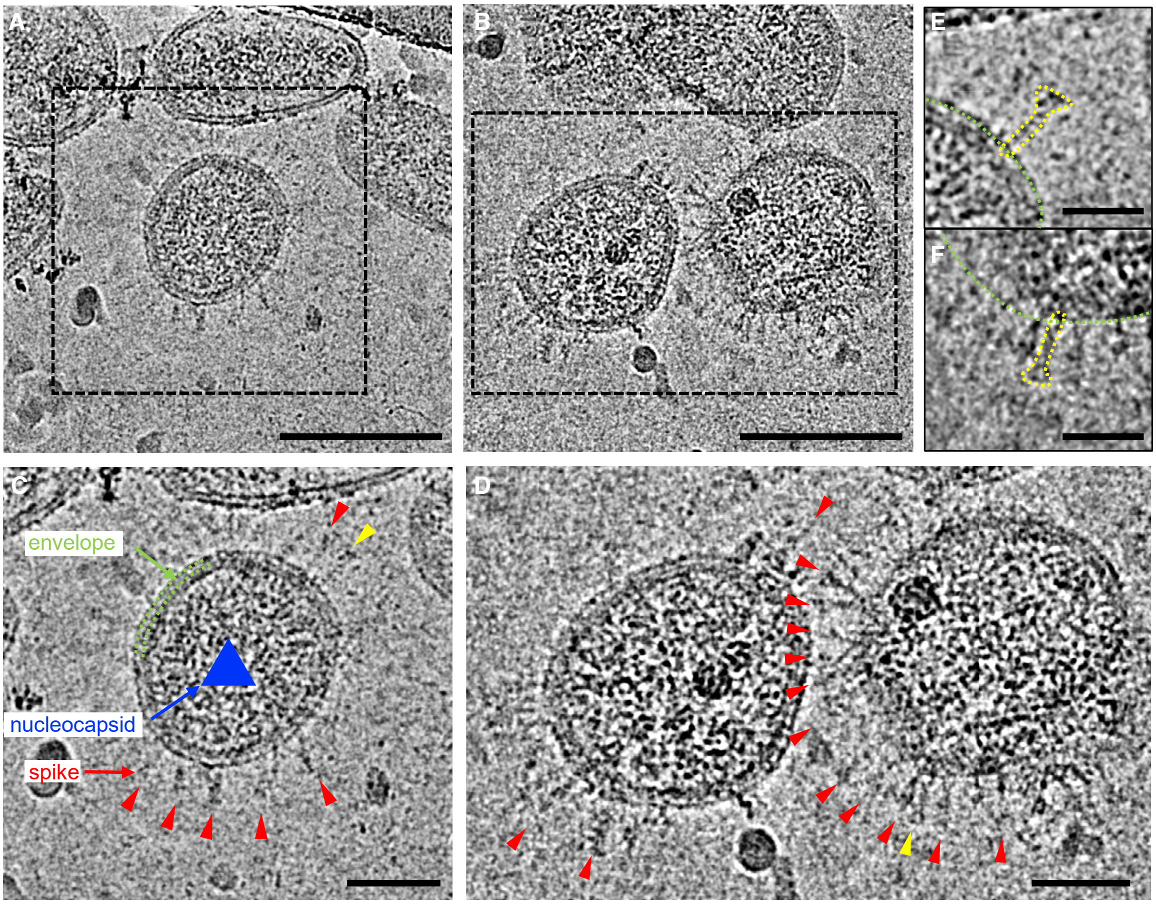

Figure 2. Cryo-EM Analysis of SARS-CoV-2

(A and B) Representative cryo-EM images of purified inactivated SARS-CoV-2 virus particles.

(C and D). Zoom-in views of the boxed virions in (A and B). Envelope and nucleocapsids are indicated by green dashed lines and a blue triangle, respectively. Viral spikes are indicated by red arrowheads.

(E and F). Enlarged views of the spikes indicated by yellow arrowheads in (C and D), respectively. The shape of the spike is depicted by yellow dotted lines. Green dotted lines indicate the viral envelope. Scale bars, 100 nm in (A and B), 50 nm in (C and D), 25 nm in (E and F).

(PDB: 6M3W) (Fan et al., 2020), which shares 89.97% sequence identity with SARS-CoV-2 at the S2 region (Figure S4), fit well into the subtomogram averaged density map. A significant portion of density at the bottom of the spike close to the membrane was not modeled (Figure 3C). This region could account for about 224 residues that were not resolved in the SARS-CoV structure (Figure S4). Protrusions on the side and top of the map are consistent with glycosylation moieties, in particular at the N1194 and N1173 sites (Figure 3D). It should be noted that these glycan densities are more extensive than what was modeled in PDB: 6M3W (just one sugar) (Fan et al., 2020). Glycosylation is expected to be complex and variable and often contains a chain of multiple sugars. Although the subtomogram average shows a continuous density that includes the viral membrane, the lipid membrane bilayer is not well resolved. This indicates that the postfusion spikes are flexible and have some confined variable orientations relative to the viral membrane. Due to the limited number of prefusion spikes available on the virus particles, a high-resolution subtomogram average of the prefusion spike was not obtained.

DISCUSSION

The relationship between receptor binding, priming proteolytic cleavage, and the pre- to postfusion spike transition is still not clear. Here, we have shown that isolated SARS-CoV-2 viruses treated with the inactivating agent β-propiolactone exhibit most of the spikes in the postfusion conformation, forming clusters. The density map of postfusion spikes derived from *in situ* subtomogram averaging of native virus particles shows a structure similar to that of the SARS-CoV S2 spikes previously determined by cryo-EM single-particle analysis of recombinant proteins. The anticipated glycosylation sites in the subtomogram average of SARS-CoV-2 postfusion spikes match well with the atomic model of the SARS-CoV S2 spike.

The use of chemically inactivated pathogens is one of the most common vaccine strategies. It has a good track record of generating long-lasting immunity for many different viral diseases, such as flu, polio, and yellow fever. Nonetheless, this strategy is not universally suited to all viruses and can even have disastrous consequences if a molecular and structural understanding

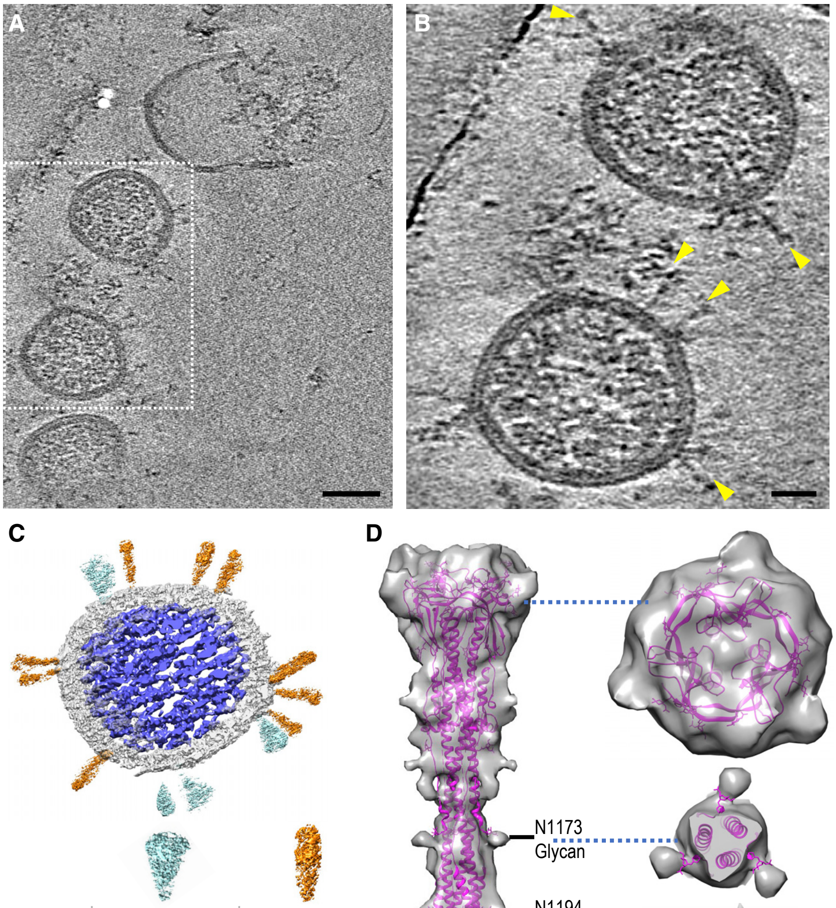

	Prefusion	Postfusion
Proportion	25.6% (32/125)	74.4% (93/125)
95% CI	18.7% - 33.9%	66% - 81.3%

Figure 3. Cryo-ET and Subtomogram Averaging of SARS-CoV-2 Postfusion Spike

(A) Tomographic slice of inactivated SARS-CoV-2 viruses.

(B) Enlarged view of the boxed region in (A). Viral spikes are indicated by yellow arrowheads.

(C) Segmentation of a representative virus particle, showing the prefusion spikes in cyan, postfusion spikes in orange, membrane in light gray, and ribonucleoprotein and RNA genome complex in blue. The distribution of prefusion and postfusion spikes and the 95% confidence interval are listed below.

(D) Density map of the postfusion SARS-CoV-2 spike fitted with a SARS-CoV model (PDB: 6M3W) (Fan et al., 2020). The unmodeled density at the bottom could account for 224 residues not resolved in PDB: 6M3W. Two glycan sites are labeled. Right side shows sectional views at the positions marked by the blue dashed lines. Scale bars, 50 nm in (A), 20 nm in (B).

of the antigen is lacking. One such unfortunate example is the formalin-inactivated respiratory syncytial virus (FI-RSV) vaccine trial of the 1960s, which led to enhancement of disease symptoms in vaccinated children after natural exposure to RSV, with two fatal cases. The molecular mechanism of this negative effect of the RSV vaccine was not fully understood until the structures of formalin-inactivated RSV were determined (McLellan et al., 2013). The RSV spike is formed by the F protein, a class I fusion protein similar to coronaviruses' S protein, and can adopt a prefusion or a postfusion state. Structural studies revealed that one contributing factor to the vaccine failure was that the prefusion state of the RSV spike was absent and the postfusion state was primarily represented in the FI-RSV vaccine formula (Killikelly et al., 2016; McLellan et al., 2013). This highlights the need to understand the differential roles of neutralizing and non-neutralizing antibodies elicited by vaccines and the challenge of avoiding antibody-dependent enhancement.

β-propiolactone is the chemical inactivating agent successfully used in rabies and other vaccines. Here we have shown that β-propiolactone-treated SARS-CoV-2 viruses exhibit most of their spikes in the postfusion conformation. It is possible that β-propiolactone could induce this conformation change, yet we cannot rule out the effect of purification and concentration procedures. Most COVID-19 vaccine candidates rely on the S protein as its antigen, since this is the primary exposed protein on the surface of the SARS-CoV-2 viral particle. As of July 7, 2020, 21 COVID-19 vaccine candidates were in clinical evaluation, 4 of them utilizing inactivated viruses (WHO, 2020a). β-propiolactone was used in three of four candidates as the inactivation reagent (Chen et al., 2020; Gao et al., 2020; Wrapp et al., 2020a; Xia et al., 2020) (Table S3). One of the vaccine candidates, PiCoVacc, was purified and inactivated the same way as in this study, and not surprisingly also showed substantial postfusion spikes, even though a prefusion state was incorrectly claimed (Gao et al., 2020). Therefore, structural studies, particularly on the conformational state of the viral spike *in situ* in intact virus particles, are paramount for these vaccine candidates, especially when the antigenicity of a vaccinal antigen is not predictive of the protective immunity elicited by it, as the FI-RSV trial shows.

STAR★METHODS

Detailed methods are provided in the online version of this paper and include the following:

- KEY RESOURCES TABLE
- RESOURCE AVAILABILITY
  - ○ Lead Contact
  - ○ Materials Availability
  - ○ Data and Code Availability
- EXPERIMENTAL MODEL AND SUBJECT DETAILS

- Ethics Statement
- Cell Lines and Viruses
- METHOD DETAILS
  - Sample Collection and Virus Isolation
  - qRT-PCR
  - Indirect Immunofluorescence Assay (IFA)
  - Enzyme Linked Immunosorbent Assay (ELISA)
  - Virus Preparation
  - Viral Genome Sequence and Phylogenetic Analyses
  - Negative Stain EM Imaging
  - Cryo-EM Imaging
  - Cryo-ET Data Acquisition
  - Cryo-ET Image Processing and Subtomogram Averaging
- QUANTIFICATION AND STATISTICAL ANALYSIS

SUPPLEMENTAL INFORMATION

Supplemental Information can be found online at https://doi.org/10.1016/j.str.2020.10.001.

ACKNOWLEDGMENTS

We acknowledge the work and contribution of all the health providers from the Third People's Hospital of Shenzhen. We sincerely thank both the cryo-EM center and the BSL-3 laboratory of the Second Affiliated Hospital, School of Medicine, in South University of Science and Technology for providing the facilities and technical support. We thank J. Dong, C. Freeman, and R. Esnouf for computer system support. We also thank Professors Dongfeng Gu and Mingzhao Xing and Ms. Anna Rackley for their valuable advice and reading of the manuscript.

This research was supported by the Science and Technology Innovation Committee of Shenzhen Municipality (202002073000002 and JSGG 20200207155251653) and National Institutes of Health grant P50AI150481 (P.Z.), UK Wellcome Trust Investigator Award 206422/Z/17/Z (P.Z.), and UK Biotechnology and Biological Sciences Research Council grant BB/S003339/1 (P.Z.).

AUTHOR CONTRIBUTIONS

Z.Z., L.L., P.W., and P.Z. conceived and designed the research. C.L., Y.G., T.N., C.C.L., and S.X. collected EM, cryo-EM, and cryo-ET data. C.L., L.M., Y.G., T.N., J.L., Y.Z., and Z.L. analyzed and interpreted cryo-EM and cryo-ET data. P.Z. and L.M. performed model fitting and analysis. Y.Y. and C.S. contributed to the virion isolation and purification. B.J. contributed to the antibody production. X.T. and J.W. contributed to the technical support of virus culture. W.J. contributed to the BSL-3 support. X.M. and J.W. contributed to the technical support of transmission electron microscope operation. Y.L. and J.Y. contributed to the clinical data collection. C.L., L.M., Y.Y., C.S., P.W., and P.Z. wrote the manuscript with the support and approval of all the authors.

DECLARATION OF INTERESTS

The authors declare no competing financial or non-financial interests.

Received: July 20, 2020
Revised: August 23, 2020
Accepted: September 30, 2020
Published: October 20, 2020

REFERENCES

Castaño-Díez, D., Kudryashev, M., Arheit, M., and Stahlberg, H. (2012). Dynamo: a flexible, user-friendly development tool for subtomogram averaging of cryo-EM data in high-performance computing environments. J Struct Biol. *178*, 139–151.

Chen, H., Xie, Z., Long, R., Fan, S., Li, H., He, Z., Xu, K., Liao, Y., Wang, L., Zhang, Y., et al. (2020). A valid protective immune response elicited in rhesus macaques by an inactivated vaccine is capable of defending against SARS-CoV-2 infection. bioRxiv.

Fan, X., Cao, D., Kong, L., and Zhang, X. (2020). Cryo-EM analysis of the post-fusion structure of the SARS-CoV spike glycoprotein. Nat. Commun. *11*, 3618.

Gao, Q., Bao, L., Mao, H., Wang, L., Xu, K., Yang, M., Li, Y., Zhu, L., Wang, N., Lv, Z., et al. (2020). Development of an inactivated vaccine candidate for SARS-CoV-2. Science *369*, 77–81.

Hagen, W.J.H., Wan, W., and Briggs, J.A.G. (2017). Implementation of a cryo-electron tomography tilt-scheme optimized for high resolution subtomogram averaging. J. Struct. Biol. *197*, 191–198.

Himes, B.A., and Zhang, P. (2018). emClarity: software for high-resolution cryo-electron tomography and subtomogram averaging. Nat. Methods *15V*, 955–961.

Hoffmann, M., Kleine-Weber, H., Schroeder, S., Krüger, N., Herrler, T., Erichsen, S., Schiergens, T.S., Herrler, G., Wu, N.H., Nitsche, A., et al. (2020). SARS-CoV-2 cell entry depends on ACE2 and TMPRSS2 and is blocked by a clinically proven protease inhibitor. Cell *181*, 271–280.e8.

Ju, B., Zhang, Q., Ge, J., Wang, R., Sun, J., Ge, X., Yu, J., Shan, S., Zhou, B., Song, S., et al. (2020). Human neutralizing antibodies elicited by SARS-CoV-2 infection. Nature *584*, 115–119.

Killikelly, A.M., Kanekiyo, M., and Graham, B.S. (2016). Pre-fusion F is absent on the surface of formalin-inactivated respiratory syncytial virus. Sci. Rep. *6*, 34108.

Kremer, J.R., Mastronarde, D.N., and McIntosh, J.R. (1996). Computer visualization of three-dimensional image data using IMOD. J. Struct. Biol. *116*, 71–76.

Lan, J., Ge, J., Yu, J., Shan, S., Zhou, H., Fan, S., Zhang, Q., Shi, X., Wang, Q., Zhang, L., et al. (2020). Structure of the SARS-CoV-2 spike receptor-binding domain bound to the ACE2 receptor. Nature *581*, 215–220.

Liu, Y., Yang, Y., Zhang, C., Huang, F., Wang, F., Yuan, J., Wang, Z., Li, J., Feng, C., Zhang, Z., et al. (2020). Clinical and biochemical indexes from 2019-nCoV infected patients linked to viral loads and lung injury. Sci. China Life Sci. *63*, 364–374.

Mastronarde, D.N. (2005). Automated electron microscope tomography using robust prediction of specimen movements. J. Struct. Biol. *152*, 36–51.

McLellan, J.S., Chen, M., Leung, S., Graepel, K.W., Du, X., Yang, Y., Zhou, T., Baxa, U., Yasuda, E., Beaumont, T., et al. (2013). Structure of RSV fusion glycoprotein trimer bound to a prefusion-specific neutralizing antibody. Science *340*, 1113–1117.

Ni, T., Gerard, S., Zhao, G., Dent, K., Ning, J., Zhou, J., Shi, J., Anderson-Daniels, J., Li, W., Jang, S., et al. (2020). Intrinsic curvature of the HIV-1 CA hexamer underlies capsid topology and interaction with cyclophilin A. Nat. Struct. Mol. Biol. *27*, 855–862.

Perlman, S., and Netland, J. (2009). Coronaviruses post-SARS: update on replication and pathogenesis. Nat. Rev. Microbiol. *7*, 439–450.

Pettersen, E.F., Goddard, T.D., Huang, C.C., Couch, G.S., Greenblatt, D.M., Meng, E.C., and Ferrin, T.E. (2004). UCSF Chimera–a visualization system for exploratory research and analysis. J. Comput. Chem. *25*, 1605–1612.

Song, W., Gui, M., Wang, X., and Xiang, Y. (2018). Cryo-EM structure of the SARS coronavirus spike glycoprotein in complex with its host cell receptor ACE2. PLoS Pathog. *14*, e1007236.

Sutton, G., Sun, D., Fu, X., Kotecha, A., Hecksel, C.W., Clare, D.K., Zhang, P., Stuart, D.I., and Boyce, M. (2020). Assembly intermediates of orthoreovirus captured in the cell. Nat. Commun. *11*, 4445.

Viruses, C.S.G.o.t.I.C.o.T.o. (2020). The species Severe acute respiratory syndrome-related coronavirus: classifying 2019-nCoV and naming it SARS-CoV-2. Nat. Microbiol. *5*, 536–544.

Walls, A.C., Tortorici, M.A., Snijder, J., Xiong, X., Bosch, B.J., Rey, F.A., and Veesler, D. (2017). Tectonic conformational changes of a coronavirus spike

glycoprotein promote membrane fusion. Proc. Natl. Acad. Sci. U S A *114*, 11157–11162.

Wang, C., Horby, P.W., Hayden, F.G., and Gao, G.F. (2020). A novel coronavirus outbreak of global health concern. Lancet *395*, 470–473.

WHO. (2020a). Draft Landscape of COVID-19 Candidate Vaccines (World Health Organization).

WHO. (2020b). WHO Coronavirus Disease (COVID-19) Dashboard (World Health Organization).

Wrapp, D., Wang, N., Corbett, K.S., Goldsmith, J.A., Hsieh, C.-L., Abiona, O., Graham, B.S., and McLellan, J.S. (2020a). Cryo-EM Structure of the 2019-nCoV Spike in the Prefusion Conformation, p. eabb2507.

Wu, Y., Wang, F., Shen, C., Peng, W., Li, D., Zhao, C., Li, Z., Li, S., Bi, Y., Yang, Y., et al. (2020). A noncompeting pair of human neutralizing antibodies block COVID-19 virus binding to its receptor ACE2. Science *368*, 1274–1278.

Xia, S., Duan, K., Zhang, Y., Zhao, D., Zhang, H., Xie, Z., Li, X., Peng, C., Zhang, Y., Zhang, W., et al. (2020). Effect of an inactivated vaccine against SARS-CoV-2 on safety and immunogenicity outcomes: interim analysis of 2 randomized clinical trials. JAMA *324*, 1–10.

Yan, R., Zhang, Y., Li, Y., Xia, L., Guo, Y., and Zhou, Q. (2020). Structural basis for the recognition of the SARS-CoV-2 by full-length human ACE2. Science *367*, 1444–1448.

Yang, Y., Shen, C., Li, J., Zou, R., Wong, G., Peng, L., Yang, L., Fang, S., Li, X., Wu, W., et al. (2019). Clinical and virological characteristics of human infections with H7N9 avian influenza virus in Shenzhen, China, 2013-2017. J. Infect. *79*, 389–399.

Yang, Y., Ye, F., Zhu, N., Wang, W., Deng, Y., Zhao, Z., and Tan, W. (2015). Middle East respiratory syndrome coronavirus ORF4b protein inhibits type I interferon production through both cytoplasmic and nuclear targets. Sci. Rep. *5*, 17554.

Yang, Y., Zhang, L., Geng, H., Deng, Y., Huang, B., Guo, Y., Zhao, Z., and Tan, W. (2013). The structural and accessory proteins M, ORF 4a, ORF 4b, and ORF 5 of Middle East respiratory syndrome coronavirus (MERS-CoV) are potent interferon antagonists. Protein Cell *4*, 951–961.

Zhang, P. (2019). Advances in cryo-electron tomography and subtomogram averaging and classification. Curr. Opin. Struct. Biol. *58*, 249–258.

Zheng, S.Q., Palovcak, E., Armache, J.P., Verba, K.A., Cheng, Y., and Agard, D.A. (2017). MotionCor2: anisotropic correction of beam-induced motion for improved cryo-electron microscopy. Nat. Methods *14*, 331–332.

Zhu, N., Zhang, D., Wang, W., Li, X., Yang, B., Song, J., Zhao, X., Huang, B., Shi, W., Lu, R., et al. (2020). A novel coronavirus from patients with pneumonia in China, 2019. N. Engl. J. Med. *382*, 727–733.

STAR★METHODS

KEY RESOURCES TABLE

REAGENT or RESOURCE	SOURCE	IDENTIFIER
**Antibodies**		
HRP-conjugated Goat anti-human IgG	Sangon Biotech	Cat.# D110150-0001; RRID: AB_2876788
**Bacterial and Virus Strains**		
BetaCoV/Shenzhen/SZTH-003/2020	Shenzhen Third People's Hospital	EPI ISL 406594
**Biological Samples**		
Fetal Bovine Serum	Corning	Cat.# 35-076-CV
BetaCoV/Shenzhen/SZTH-003/2020	This manuscript	N/A
**Chemicals, Peptides, and Recombinant Proteins**		
PBST	Sangon Biotech	Cat.# C006162-0250
Block Buffer in PBS with Non-Fat Milk	Sangon Biotech	Cat.# C520013-0500
Sodium chloride	Sangon Biotech	Cat.# A610476-0001
1M Hepes Solution	Sangon Biotech	Cat.# E607018-0100
Potassium tartrate	Sangon Biotech	Cat.# A600784-0500
Glycerol	Sangon Biotech	Cat.# A100854-0100
DMEM	GIBCO	Cat.# C11995500BT
0.25% Typsin-EDTA	GIBCO	Cat.# 25200-056
Pen-strep	GIBCO	Cat.# 15140-122
Phosphotungstic Acid	Sigma-Aldrich	Cat.# 79690-25
**Critical Commercial Assays**		
TMB Chromogen Solution (for ELISA)	Sangon Biotech	Cat.# E661007-0100
ELISA Stopping Solution	Sangon Biotech	Cat.# E661006-0200
QIAamp Viral RNA Mini Kit	Qiagen	Cat.# 52906
2019-nCoV qPCR kit	BioGerm	Cat.# ZC-HX-201-2
ELISA Plate	Sangon Biotech	Cat.# F605031-0001
**Deposited Data**		
Genome sequence of isolated virus strain "BetaCoV/Shenzhen/SZTH-003/2020"	This paper	Global Initiative on Sharing Avian Flu Data (GISAID) with an access number of EPI_ISL_406594
Post-fusion structure of SARS-CoV spike glycoprotein	(Fan et al.,2020)	PDB ID: 6M3W
Postfusion SARS-CoV-2 Spike Protein	This paper	EMD-11627
**Experimental Models: Cell Lines**		
Vero Cell Lines	ATCC	Cat.# CCL-81
**Software and Algorithms**		
7500 Software v2.0.5	Applied Biosystems	NA
MOTIONCOR2	(Zheng et al.,2017)	https://emcore.ucsf.edu/ucsf-software
IMOD	(Kremer et al.,1996)	https://bio3d.colorado.edu/imod/
Dynamo	(Castaño-Díez et al.,2012)	https://wiki.dynamo.biozentrum.unibas.ch/w/index.php/Main_Page
MATLAB	MathWorks	https://www.mathworks.com/products/matlab.html
emClarity	(Himes and Zhang, 2018)	https://github.com/bHimes/emClarity/wiki
UCSF CHIMERA 1.13	(Pettersen et al., 2004)	https://www.cgl.ucsf.edu/chimera/
**Other**		
Continuous Carbon Layer	Electron microscopy China	N/A
Quantifoil grid (R2/2) Mo 300 mesh	Quantifoil Micro Tools GmbH	N/A

RESOURCE AVAILABILITY

Lead Contact

Further information and requests should be directed and will be fulfilled by the Lead Contact, Prof. Peijun Zhang, (peijun@strubi.ox.ac.uk).

Materials Availability

Viral strain is available from Drs Zheng Zhang (zhangzheng1975@aliyun.com) and Lei Liu (liulei3322@aliyun.com) with a completed Materials and Transfer Agreement.

Data and Code Availability

The genome sequence of isolated virus was submitted to the Global Initiative on Sharing Avian Flu Data (GISAID) with an access number of EPI_ISL_406594 and designated as "BetaCoV/Shenzhen/SZTH-003/2020".

CryoEM density map for postfusion SARS-CoV-2 Spike Protein is deposited at the EMDB under accession code EMD-11627.

EXPERIMENTAL MODEL AND SUBJECT DETAILS

Ethics Statement

The research received approval from the Research Ethics Committee of Shenzhen Third People's Hospital, China (approval number: 2020-038). The Research Ethics Committee waived the requirement for informed consent before the study started because of the urgent need to collect epidemiological and clinical data. We analysed the data anonymously.

Cell Lines and Viruses

African green monkey kidney Vero cell (Female) (ATCC, CCL-81) were obtained from ATCC and maintained in Dulbecco's minimal essential medium (DMEM)(GIBCO) supplemented with 10% fetal bovine serum (FBS)(Corning) and penicillin (100U/ml)-streptomycin (100mg/ml)(GIBCO). Cell line has not been authenticated.

Patient-derived SARS-CoV-2 isolate BetaCoV/Shenzhen/SZTH-003/2020 (EPI_ISL_406594) was isolated the BALF sample of the COVID-19 patient using Vero cell in biosafety level 3 (BSL-3) laboratory.

METHOD DETAILS

Sample Collection and Virus Isolation

The Bronchoalveolar Lavage Fluid (BALF) was collected from the patient at 1 day after admission. Vero cells were used for the virus isolation in the biosafety level 3 (BSL-3) laboratory. The BALF sample was centrifuged at 5, 000 rpm at 4°C for 5 minutes, and then 200 μl supernatant was added to monolayer of cell in 6 well plates and incubated at 37°C and 5% CO_2 for 1 hour. Then cells were washed with PBS 3 times, and fresh DMEM containing 2% fetal bovine serum (FBS) and 1% penicillin streptomycin (PS) was added to cell culture. Cells were maintained at 37°C and 5% CO_2, and CPEs were monitored daily with light microscopy. Meanwhile viral RNAs were detected at 3 and 5 dpi using qRT-PCR to monitor the replication of virus, and the supernatant was harvested at 6 dpi.

qRT-PCR

Viral RNAs from BALF and cell culture supernatant were extracted using the QIAamp RNA Viral Kit (Qiagen, Heiden, Germany). Then quantitative reverse transcription polymerase chain reaction (qRT-PCR) was performed using a commercial kit specific for 2019-nCoV detection targeting the ORF lab and N genes (BioGerm, Ltd., Shanghai, China) as reported previously (Liu et al., 2020). The amplification conditions were: Incubation at 50°C for 10min,Taq activation for 5 min at 95°C, followed by 40 cycles of amplification comprising denaturation for 10 sec at 95°C, annealing and primer extension for 40 sec at 55°C. The specimens were considered positive if the Ct value was 37.0 or lower, negative if the result were undetermined. Specimens with a Ct value higher than 37.0 were repeated. The specimen was considered positive if the repeated results were the same as the initial result and between 37 and 40. If the repeated Ct was undetectable, the specimen was considered negative.

Indirect Immunofluorescence Assay (IFA)

IFA was done as described previously (Yang et al., 2013, 2015). Vero cells were fixed in 4% formaldehyde at 48 hours post infection. Then cells were permeabilized in 0.5% Triton X-100, blocked in 5% BSA in PBS, and then probed with the plasma of this patient or healthy control at a dilution of 1:500 for 1 h at room temperature. The cells were washed three times with PBS and then incubated with either goat anti-human IgG conjugated with Alexa fluor 488 at a dilution of 1:500 for 1 h (Invitrogen, Carlsbad, CA). The cells were then washed and stained with hoechest-33342 (Invitrogen, Carlsbad, CA) to detect nuclei. Fluorescence images were obtained and analysed using EVOS FL Auto Imaging System (Invitrogen, Carlsbad, CA).

CellPress
OPEN ACCESS

Enzyme Linked Immunosorbent Assay (ELISA)

Microtiter plates (Sangon Biotech) were coated overnight at 4°C with 2.5 × 10^3 $TCID_{50}$/well/100 μl purified and inactivated SARS-CoV-2 particles. The plates were washed twice with PBS containing 0.1% v/v Tween-20 (PBST) and blocked with blocking solution (PBS containing 2% w/v non-fat dry milk) for 2 hours at 37°C. The plates were then washed with PBST. The plasma was diluted to 1000-fold and human monoclonal antibody specific to SARS-CoV-2-RBD generated by our laboratory were diluted to 200 μg/ml into PBS as initial concentration, and serial 3-fold dilutions of sera was added to the wells and incubated at 37°C for 60 minutes. After three washes, 100 μl of horseradish peroxidase (HRP)-conjugated goat anti-human IgG antibody solution (Sangon Biotech) was added to each well and incubated at 37°C for 60 minutes. After washing, 100 μl of tetramethylbenzidine (TMB) substrate (Sangon Biotech) was added at room temperature in the dark. After 15 minutes, the reaction was stopped with a 2M H2SO4 solution. The absorbance was measured at 450 nm. All data points represent a single measurement. The ELISA titers were determined by endpoint dilution.

Virus Preparation

Virus was amplified in Vero cell in an incubator at 37°C and 5% CO_2 for 96 hours and the viral load was determined by qPCR assay. The harvested 400ml virus supernatant with a virus titer of about 1.08 × 10^5 $TCID_{50}$/ml (genome equivalent) was firstly inactivated by adding 0.05% (v/v) β-propiolactone and incubating at 4°C for 36 hours.

Then the inactivated viruses were pelleted by centrifugation at 85,000 g, 4°C using a SW28 rotor. The pellet was dissolved at 4°C overnight in buffered saline (0.15M NaCl, 20mM HEPES, pH 6.8). The 3 ml of the suspended virus was layered on top of linear gradient of 40% (w/w) potassium tartrate, 20mM HEPES, pH 7.4 (bottom) to 15% (w/w) glycerol, 20mM HEPES, pH 7.4 (top) and subjected to isopycnic centrifugation in SW40 rotor at 85,000 g, 4°C for 3 hours. The resulting milky band of virus was diluted with buffered saline and pelleted by centrifugation using an SW40 rotor at 85,000, 4°C for another 3 hours. Finally, the pellet was allowed to dissolve at 4°C overnight in 50 μl of buffered saline. Six independent virus samples were prepared and analysed in the following work. All following work was not blinded. Randomization, subject inclusion and/or exclusion criteria and sample size estimation do not apply to this sample.

Viral Genome Sequence and Phylogenetic Analyses

The extracted viral RNAs were subjected for next generation sequencing using the Illumina sequencing platform. The assembled sequence was further analyzed together with other genomes of SARS-CoV-2 downloaded from National Center for Biotechnology Information (NCBI) database. The phylogenetic analyses were completed as previously reported(Yang et al., 2019). Software Muscle was used for multiple sequence alignment. The phylogenetic analysis was performed using RAxML, 1000 bootstrap replicates were run.

Negative Stain EM Imaging

An aliquot of 3μl purified sample was applied to a glow-discharged grid with continuous carbon layer (Electron microscopy China, China) for 1min. Grid was then blotted by filter paper to absorb excrescent sample. The grid was then stained with 3%(w/w) phosphotungstic acid for 1min. Redundant liquid was absorbed using filter paper. The grid was transferred to a Talos 120C transmission electron microscope (Thermo Fisher Scientific) performed at the 120 kV. Data was collected at a nominal magnification of 45000 ×, corresponding to a pixel size of 3.5 Å/pixel with a Ceta 16M CMOS detector (Thermo Fisher Scientific).

Cryo-EM Imaging

For cryo-EM sample preparation, 3μl of sample was placed on a glow-discharged Quantifoil grid (R1.2/1.3). The grid was blotted using Vitrobot Mark IV (Thermo Fisher Scientific). Frozen grid was then transferred to a Titan Krios transmission electron microscope (Thermo Fisher Scientific) operated at 300 kV and images were recorded by a Falcon III direct electron detector (Thermo Fisher Scientific). Image stacks with 16 frames were collected in linear mode by EPU at a nominal magnification of 59000 x, corresponding to a pixel size of 1.14 Å/pixel. Total dose was estimated at about 60 $e^-/Å^2$.

Cryo-ET Data Acquisition

Tilt-series were acquired on a Gatan K2-Summit detector in super-resolution mode using an FEI Titan Krios operating at 300 kV equipped with an energy filter (slit width 20 eV; GIF Quantum LS, Gatan). Tilt-series were recorded at a nominal magnification of 105,000x, corresponding to a calibrated pixel size of 0.7 Å. A dose-symmetric scheme(Hagen et al., 2017) was used to collect tilt-series from -60° to 60° at a step size of 3° using SerialEM software(Mastronarde, 2005). At each tilt, a movie stack consisting of 10 frames was recorded with a set dose rate of 3 $e^-/Å^2$/sec. Tilt-series were collected at a range of nominal defocus between -1.5 and -4.5 μm and a target total dose of 120 $e^-/Å^2$ was applied over the entire series.

Cryo-ET Image Processing and Subtomogram Averaging

Frames were motion-corrected and Fourier-cropped to a final pixel size of 1.4 Å using MotionCor2(Zheng et al., 2017). Tilt-series were aligned using eTomo using gold fiducials. Tomographic reconstructions were produced using tilt-series binned by a factor of 6 and using SIRT-like filter equivalent to 10 iterations in order to enhance contrast. Postfusion spike positions were manually picked in IMOD(Kremer et al., 1996). To acquire initial particle orientations, the tomograms were imported to Dynamo(Castaño-Díez et al.,

2012) and oriented surface models were hand-drawn so that model points were oriented normal to the viral membrane. In-house MATLAB scripts were used to copy the orientation from the closest membrane point to the manually picked postfusion spikes points. 956 subtomograms were cropped, and iteratively aligned and averaged using Dynamo and emClarity(Pettersen et al., 2004), first using bin 6 boxes, and later using bin 2 boxes. Images and atomic-model fittings of PDB 6M3W were produced in UCSF Chimera(Pettersen et al., 2004).

QUANTIFICATION AND STATISTICAL ANALYSIS

Spike distributions were determined after visual inspection and manually counting pre and postfusion spikes in 19 randomly selected viral particles (for a total of 125 spikes). 95% Confidence intervals of pre and postfusion spike proportions were calculated by the modified Wald method using the GraphPad QuickCalcs Web site: https://www.graphpad.com/quickcalcs/confInterval2/ (accessed in September 2020).

Viral particle diameter was measured in IMOD(Kremer et al., 1996). Measurements plotting and statistical analysis was performed using GraphPad Prism. Data are presented as mean ± SEM.

The investigators were not blinded to allocation during experiments and outcome assessment.

Structure, Volume *28*

Supplemental Information

The Architecture of Inactivated SARS-CoV-2 with Postfusion Spikes Revealed by Cryo-EM and Cryo-ET

Chuang Liu, Luiza Mendonça, Yang Yang, Yuanzhu Gao, Chenguang Shen, Jiwei Liu, Tao Ni, Bin Ju, Congcong Liu, Xian Tang, Jinli Wei, Xiaomin Ma, Yanan Zhu, Weilong Liu, Shuman Xu, Yingxia Liu, Jing Yuan, Jing Wu, Zheng Liu, Zheng Zhang, Lei Liu, Peiyi Wang, and Peijun Zhang

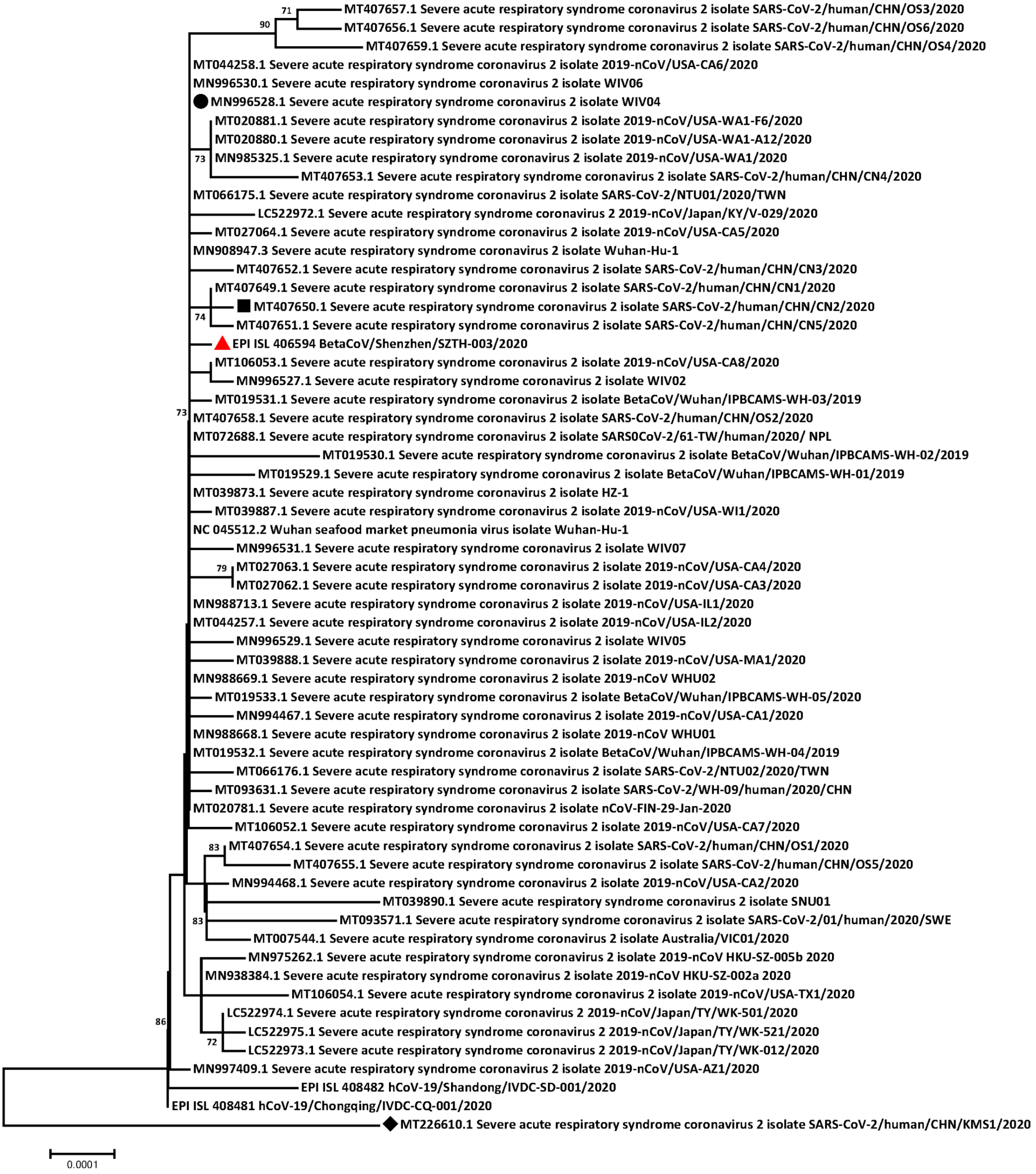

Figure S1 | Phylogenetic characteristics of BetaCoV/Shenzhen/SZTH-003/2020. This isolate was indicated with a triangle. Red triangle, this study; Black dot, WIV04; black squar, CN2; black diamond, UCC91-02. Related to Figure 1.

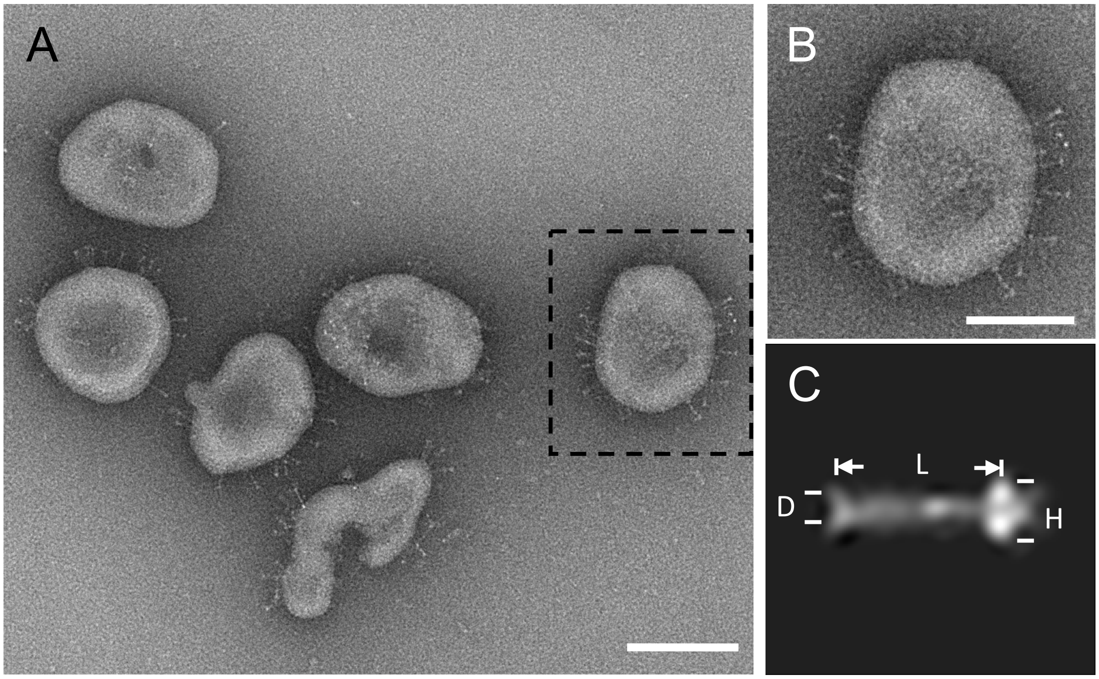

Figure S2 | Characterization of purified SARS-CoV-2 by negative staining EM. (A) Image of negative stained SARS-CoV-2. Nail-like spikes can be clearly seen. (B) Enlarged view of virion boxed in (A). (C) Averaged 2D projection of SARS-CoV-2 nail-shaped spike (Song et al., 2018). The length (L), diameter of stem (D) and spike's head (H) are 23nm, 4nm and 7nm, respectively. Scale bars, 100 nm in A, 50 nm in B. Related to Figure 2.

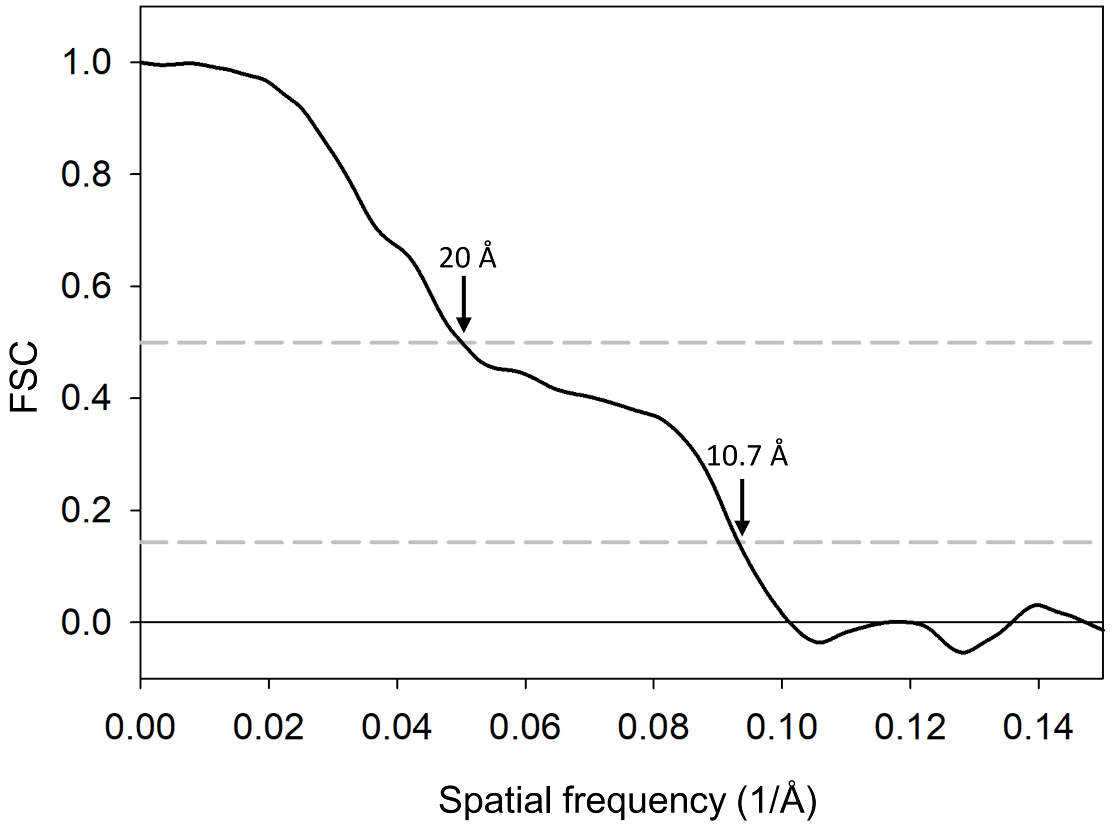

Figure S3 | Fourier-shell-correlation (FSC) plot of postfusion spike subtomogram averaged density maps. The two dashed lines mark the FSC value of 0.5 and 0.143. The resolution is 20 Å at FSC of 0.5 and 10.7 Å at FSC of 0.143. Related to Figure 3.

```
Uniprot|Sequence name


P0DTC2|SPIKE_SARS2      NNSYECDIPIGAGICASYQTQTNSPRRARSVASQSIIAYTMSLGAENSVAYSNNSIAIPT   716
P59594|SPIKE_CVHSA      DTSYECDIPIGAGICASYHTVSL----LRSTSQKSIVAYTMSLGADSSIAYSNNTIAIPT   698
                        :.****************:* :      **.:.:**:********:.*:*****:*****

P0DTC2|SPIKE_SARS2      NFTISVTTEILPVSMTKTSVDCTMYICGDSTECSNLLLQYGSFCTQLNRALTGIAVEQDK   776
P59594|SPIKE_CVHSA      NFSISITTEVMPVSMAKTSVDCNMYICGDSTECANLLLQYGSFCTQLNRALSGIAAEQDR   758
                        **:**:***::****:******.**********:*****************:***.***:

P0DTC2|SPIKE_SARS2      NTQEVFAQVKQIYKTPPIKDFGGFNFSQILPDPSKPSKRSFIEDLLFNKVTLADAGFIKQ   836
P59594|SPIKE_CVHSA      NTREVFAQVKQMYKTPTLKYFGGFNFSQILPDPLKPTKRSFIEDLLFNKVTLADAGFMKQ   818
                        **:********:**** :* ************* **:********************:**

P0DTC2|SPIKE_SARS2      YGDCLGDIAARDLICAQKFNGLTVLPPLLTDEMIAQYTSALLAGTITSGWTFGAGAALQI   896
P59594|SPIKE_CVHSA      YGECLGDINARDLICAQKFNGLTVLPPLLTDDMIAAYTAALVSGTATAGWTFGAGAALQI   878
                        **:***** **********************:*** **:**::** *:************

P0DTC2|SPIKE_SARS2      PFAMQMAYRFNGIGVTQNVLYENQKLIANQFNSAIGKIQDSLSSTASALGKLQDVVNQNA   956
P59594|SPIKE_CVHSA      PFAMQMAYRFNGIGVTQNVLYENQKQIANQFNKAISQIQESLTTTSTALGKLQDVVNQNA   938
                        ************************* ******.**.:**:**::*::*************

P0DTC2|SPIKE_SARS2      QALNTLVKQLSSNFGAISSVLNDILSRLDKVEAEVQIDRLITGRLQSLQTYVTQQLIRAA   1016
P59594|SPIKE_CVHSA      QALNTLVKQLSSNFGAISSVLNDILSRLDKVEAEVQIDRLITGRLQSLQTYVTQQLIRAA   998
                        ************************************************************

P0DTC2|SPIKE_SARS2      EIRASANLAATKMSECVLGQSKRVDFCGKGYHLMSFPQSAPHGVVFLHVTYVPAQEKNFT   1076
P59594|SPIKE_CVHSA      EIRASANLAATKMSECVLGQSKRVDFCGKGYHLMSFPQAAPHGVVFLHVTYVPSQERNFT   1058
                        **************************************:**************:**:***

P0DTC2|SPIKE_SARS2      TAPAICHDGKAHFPREGVFVSNGTHWFVTQRNFYEPQIITTDNTFVSGNCDVVIGIVNNT   1136
P59594|SPIKE_CVHSA      TAPAICHEGKAYFPREGVFVFNGTSWFITQRNFFSPQIITTDNTFVSGNCDVVIGIINNT   1118
                        *******:***:******** *** **:*****:.*********************:***

P0DTC2|SPIKE_SARS2      VYDPLQPELDSFKEELDKYFKNHTSPDVDLGDISGINASVVNIQKEIDRLNEVAKNLNES   1196
P59594|SPIKE_CVHSA      VYDPLQPELDSFKEELDKYFKNHTSPDVDLGDISGINASVVNIQKEIDRLNEVAKNLNES   1178
                        ************************************************************

P0DTC2|SPIKE_SARS2      LIDLQELGKYEQYIKWPWYIWLGFIAGLIAIVMVTIMLCCMTSCCSCLKGCCSCGSCCKF   1256
P59594|SPIKE_CVHSA      LIDLQELGKYEQYIKWPWYVWLGFIAGLIAIVMVTILLCCMTSCCSCLKGACSCGSCCKF   1238
                        *******************:****************:*************.*********

P0DTC2|SPIKE_SARS2      DEDDSEPVLKGVKLHYT       1273
P59594|SPIKE_CVHSA      DEDDSEPVLKGVKLHYT       1255
                        *****************
```

Figure S4 | Sequence alignment between SARS-CoV-2 S protein (Uniprot P0DTC2, Spike_SARS2) and SARS-CoV S protein (Uniprot P59594, Spike_CHHSA), shown in S2 region. Residues in bold indicate S2 residues. Residues in red indicates residues solved in PDB 6M3W. Residues highlighted in green are the glycosylation sites. Alignment done with CLUSTAL Omega (1.2.4). Related to Figure 3.

Table S1. Clinical information of the enrolled patient. Related to Figure 1.

Characteristics	Description
Age	62
Gender	male
Disease severity	severe
Wuhan exposure history	Yes (Jan 1, 2020 – Jan 14, 2020)
Date of symptoms onset	Jan 11, 2020
Initial symptoms	Fever/Cough
Date of hospitalization	Jan 15, 2020
Co-existing chronic disease	none
Influenza A virus	-
Influenza B virus	-
Respiratory syncytial virus	-
Human Bocavirus	-
Adenovirus	-
Human metapneumovirus	-
Rhinovirus	-
HCoV-229E	-
HCoV-OC43	-
HCoV-HKU1	-
HCoV-NL63	-
MERS-CoV	-
SARS-CoV	-
Interferon atomization	Jan 16, 2020
Ribavirin	Jan 15, 2020
Methylprednisolone	no
High-flow oxygen therapy	yes
Mechanical ventilation	No
CT finding	Bilateral pneumonia
BALF sampling date	Jan 21, 2020
Outcome	Discharged

Table S2: Summary of data acquisition and image processing statistics. Related to Figure 2 and 3

<u>Data acquisition</u>	
EM equipment	Titan Krios
Voltage (kV)	300
Detector	Gatan K2
Energy filter	Gatan GIF Quantum, 20 eV slit
Super-resolution mode	Yes
Pixel size (Å)	1.4
Total electron dose ($e^-/Å^2$)	123
Dose rate (e-/$Å^2$/s)	2.55
Frame number	10
Acquisition scheme	-60/60°, 3°
Defocus range (µm)	-1.5 to -5.0
Number of tilt-series	383
Software	SerialEM
<u>Data processing</u>	
Number of tomograms	242
Number of subtomograms	956
Symmetry imposed	C3
Final resolution (Å)	20 Å at 0.5FSC, 10.7 Å at 0.143FSC
Software	IMOD/emClarity/Dynamo
EMDB number	EMD-11627

Table S3. Comparison of inactivated viruses from four vaccine candidates with this study. Related to Figure 2 and 3.

Vaccine Name	Developer	Type of Vaccine	Vaccine strain	EM images	reference
PiCoVacc	Sinovac	β-propiolactone	CN2	CryoEM, most postfusion Spikes	Gao et al, Science 2020
N/A	Wuhan Institute of Biological Products/ Sinopharm	β-propiolactone	WIV04	NO EM image	Xia et al. JAMA 2020
BBIBP-CorV	Beijing Institute of Biological Products/ Sinopharm	β-propiolactone	HB02	Negative stain most prefusion Spikes	Wang et al. Cell 2020
N/A	Institute of Medical Biology, Chinese Academy of Medical Sciences	formaldehyde	UCC91-02	Negative stain, both prefusion and post fusion	Chen et al., BioRxiv 2020
N/A	Institute for Hepatology, National Clinical Research Center for Infectious Disease, Shenzhen	β-propiolactone	SZTH-003	Negative stain and cryoEM/ET, both prefusion (26%) and postfusion (74%)	This study
